# Accuracy of plain radiographs in diagnosing biopsy-proven malignant bone lesions

**DOI:** 10.4102/sajr.v23i1.1768

**Published:** 2019-12-06

**Authors:** Erhardt Gerber, Qonita Said-Hartley, Rufkah Gamieldien, Tharbit Hartley, Sally Candy

**Affiliations:** 1 Department of Radiology, Faculty of Health Sciences, University of Cape Town, Cape Town, South Africa; 2 Cape Radiology, Cape Town, South Africa; 3 Division of Diagnostic Radiology, University of Cape Town, Groote Schuur Hospital, Cape Town, South Africa

**Keywords:** bone tumours, radiopathological correlation, plain film findings, bone tumour biopsy, histology results

## Abstract

**Background:**

The diagnosis of primary bone tumours is a three-fold approach based on a combination of clinical, radiological and histopathological findings. Radiographs form an integral part in the initial diagnosis, staging and treatment planning for the management of aggressive/malignant bone lesions. Few studies have been performed where the radiologist’s interpretation of radiographs is compared with the histopathological diagnosis.

**Objectives:**

The study aimed to determine the frequency of bone tumours at a tertiary hospital in South Africa, and, using a systematic approach, to determine the sensitivity and specificity of radiograph interpretation in the diagnosis of aggressive bone lesions, correlating with histopathology. We also determined the inter-observer agreement in radiograph interpretation, calculated the positive and negative predictive values for aggressive/malignant bone tumours and computed the cumulative effect of multiple radiological signs to determine the yield for malignant bone tumours.

**Method:**

A retrospective, descriptive and correlational study was performed, reviewing the histopathological reports of all biopsies performed on suspected aggressive bone lesions during a 3-year period from 2012 to 2014. The radiographs were interpreted by three radiologists using predetermined criteria. The sensitivity and specificity of the readers’ interpretation of the radiograph as ‘benign/non-aggressive’ or ‘aggressive/malignant’ were calculated against the histology, and the inter-rater agreement of the readers was computed using the Fleiss kappa values.

**Results:**

Of the 88 suspected ‘aggressive or malignant’ bone tumours that fulfilled the inclusion criteria, 43 were infective or malignant and 45 were benign lesions at histology. Reader sensitivity in the diagnosis of malignancy/infective bone lesions ranged from 93% to 98% with a specificity of 53% – 73%. The average kappa value of 0.43 showed moderate agreement between radiological interpretation and final histology results. The four radiological signs with the highest positive predictive values were an ill-defined border, wide zone of transition, cortical destruction and malignant periosteal reaction. The presence of all four signs on radiography had a 100% yield for a malignant bone tumour or infective lesion.

**Conclusion:**

The use of a systemic approach in the interpretation of bone lesions on radiographs yields high sensitivity but low specificity for malignancy and infection. The presence of benign bone lesions with an aggressive radiographic appearance necessitates continuation of the triple approach for the diagnosis of primary bone tumours.

## Introduction

In comparison with benign bone lesions, primary malignant lesions are much less common, found to be roughly a hundred times less frequent.^1,2^ In fact, the majority of malignant bone lesions are on account of secondary metastatic deposits.^3^

The diagnosis of primary bone tumours is based on a triple combination of clinical, radiological and histopathological findings.^4^ Relevant clinical factors include age, history of trauma, systemic symptoms, mass, malignancy or infection and correlation with clinical examination and biochemistry.^3,4^

Radiologically, despite advances in cross-sectional computed tomography (CT) and high strength magnetic resonance imaging (MRI), standard radiographic imaging remains the mainstay in the initial diagnosis, and correlates best with the final histology.^5,6^ Supplementary CT is useful for evaluating the cortex and matrix, and MRI for determining the intramedullary and soft tissue extent, as well as in assessing for skip lesions and involvement of surrounding structures.^2,7,8^

It is important that the interpreting radiologist is familiar with the features of common benign bone tumours, as appropriate recognition of these lesions avoids unnecessary additional imaging or biopsy.^9,10^ Unfortunately, benign and malignant tumours could appear similar. Some of the known mimickers include osteomyelitis, fibrous dysplasia, avulsion injuries, bone infarcts, hyperparathyroidism and osseous sarcoidosis.^11^

Undiagnosed bone lesions are classified as aggressive or benign tumours. Interpretation is possible either through a systematic analytical approach based on radiographic features or by recognition of characteristic imaging features, musculoskeletal knowledge and experience.^12,13^ Biopsy is indicated if a bone lesion has an aggressive appearance and includes an ill-defined margin, a wide zone of transition, cortical expansion and destruction, and a malignant periosteal reaction.^9^

Open biopsy is the gold standard in obtaining tissue samples for a histological diagnosis, despite risks of tumour spillage, potential morbidity, time and cost required. Specialist care in lesion management is important, particularly to avoid changes in treatment plans and unnecessary amputations related to biopsy complications.^14,15,16^ Cytologically, lesions are classified into benign, malignant and non-neoplastic, including infective and metabolic bone lesions.^2^

Previous studies that have examined pre-biopsy imaging demonstrated a poor positive predictive value for malignancy (50% – 75%).^17,18,19,20,21,22,23^ On the contrary, other studies have shown higher sensitivities and specificities when including MRI and computer-aided detection.^24,25,26,27^ Given the paucity of literature on this topic locally, this study was implemented to test a radiologist’s interpretation of plain radiographs against a histopathological diagnosis.

## Materials and methods

This was a retrospective, descriptive and correlational study comparing the assessment of malignancy on radiographic imaging with the histopathological diagnosis on biopsy.

The study population comprised all those patients (aged 13 years and older) who had undergone bone biopsy performed at Groote Schuur hospital between 01 January 2012 and 31 December 2014. Only patients with available histology were included. Patients without radiographic imaging, inadequate histology or biopsies that yielded soft tissue (non-osseous) tumours were excluded from the study.

Three general radiologists with approximately the same seniority/expertise (5-year post-graduate experience) independently reviewed the anonymised digital plain film images stored on a removable USB device; they were blinded to each other’s findings. Each radiologist categorised bone lesions as either ‘benign’, ‘aggressive/malignant’ or ‘inconclusive’ on a data collection sheet based on the following eight radiological signs: lytic lesion, ill-defined margin, wide zone of transition, malignant periosteal reaction, cortical destruction, eccentric location, multiple lesions, and absent or chondroid matrix. These signs were selected based on imaging literature.^12,13,28^ The readers were not instructed as to the number of positive/negative radiological signs that would constitute an aggressive, benign or inconclusive lesion. For each case, a final radiological diagnostic decision was generated using a majority rule from three of the principal readers: a decision of two out of three or three out of three was taken as a majority decision. The majority vote was classified as aggressive or benign. To avoid the possible devastating consequences of missing a malignant bone tumour, equivocal or inconclusive final decisions were grouped together with ‘malignant’ as positive findings in calculating sensitivity and specificity to avoid any delay in the diagnosis of a possible malignant bone tumour. ‘Non-aggressive’ final decisions were categorised as negative findings.

We calculated positive predictive value (PPV) and negative predictive value (NPV) for aggressive/malignant bone tumours, based on the eight radiological signs mentioned above. We also calculated the cumulative effect of multiple radiological signs in determining the PPV for these tumours.

Sensitivity and specificity were calculated for the readers independently and for the ‘majority vote’. Histological results were separated into benign lesions, malignant lesions and infection, and the frequencies were recorded for each category.

Results were expressed as frequencies and percentages for categorical variables. Radiopathological correlation was determined between the majority vote’s final decisions and the final histology using the kappa (κ) statistics. Inter-observer agreement was also determined for the readers’ final decisions using the kappa statistics (Fleiss kappa value). A weighted kappa value was determined for inter-rater concordance when the ‘inconclusive’ final decisions were grouped together with the ‘aggressive’ final decisions as positive and benign final decisions grouped together as negative.

## Ethical considerations

All cases were anonymised. Ethics approval was obtained from the Human Ethics Research Committee of the Faculty of Health Sciences, University of Cape Town (HREC REF: 892/2014).

## Results

The original data set comprised 138 patients who had bone biopsies performed during the study period, from 01 January 2012 to 31 December 2014. Ten patients were excluded on the basis of inadequate histological samples, 23 patients had hystologically-proven soft-tissue (non-osseous) lesions and 17 patients had no available imaging. The final sample size included 88 patients; 52% were males and 48% were females (age range 13–81 years), and 45% (40/88) were under the age of 30 years.

In the study sample, 43/88 (49%) bone lesions were found to be malignant or infective on biopsy. Of these, 31 were primary malignant bone tumours, seven were infective and five metastatic. Forty-five (51%) lesions were histologically benign. The findings are summarised in Tables 1 and 2.

**TABLE 1:** Summary of histologically malignant/aggressive of infective lesions (*n* = 43).

Malignant or aggressive	*n*
Osteosarcoma	11
Giant cell tumour	5
Metastasis	5
Tuberculosis (TB)	5
Myeloma	4
B-cell lymphoma	2
Fibrosarcoma	2
Langerhans cell histiocytosis	2
Chondrosarcoma	1
Biphasic synovial sarcoma	1
Ewings	1
Chronic osteitis	1
Acute osteomyelitis	1
Sacral chordoma	1
Undifferentiated pleomorphic sarcoma	1

**Total**	**43**

**TABLE 2:** Summary of histologically benign lesions (*n* = 45).

Benign	*n*
No malignancy	9
Giant cell tumour (with no aggressive features)	7
Osteochondroma	6
Simple bone cysts	4
Synovial chondromatosis	3
Chondroblastoma	3
Fibroma	2
Pigmented villonodular synovitis	2
Haemangioma	2
Benign cartilage neoplasm	1
Xanthomatosis	1
Well-differentiated chondroid lesion	1
Gout	1
Lipoma	1
Fibrous dysplasia	1
Subungual exostosis	1

**Total**	**45**

Radiopathological correlation was confirmed in 70/88 (79.5%) cases with an overall sensitivity of 80% and a kappa value of 0.61, demonstrating substantial agreement between radiographic interpretation and final histology.

**FIGURE 1:**
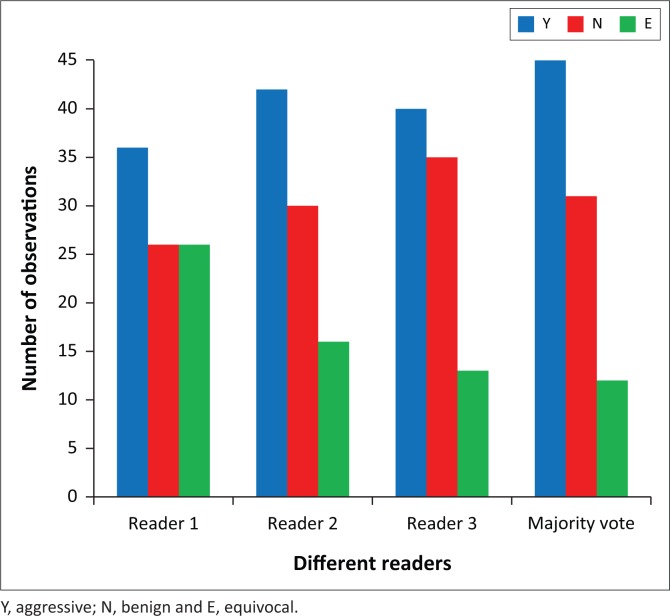
Comparison between the readers’ radiographic interpretations.

A summary of the readers’ radiological interpretations of the 88 cases is presented in Figure 1. Reader 1 categorised 36/88 lesions (41%) as aggressive/malignant, 26/88 (30%) as benign and thought that the findings were inconclusive in 26/88 (30%) cases. Combining the aggressive and inconclusive findings resulted in a sensitivity of 98% and specificity of 56%.

Similarly, combining the aggressive and inconclusive findings resulted in a sensitivity of 93% and specificity of 60% for reader 2, and a sensitivity of 95% and specificity of 73% for reader 3 (Table 3).

**TABLE 3:** Sensitivity and specificity for the interpretation of readers 1, 2 and 3.

Readers	Malignant histology (*n* = 43)	Benign histology (*n* = 45)	Total interpretations (*n* = 88)
**Reader 1**			
Positive/inconclusive radiograph interpretation	42	20	62
Negative radiograph interpretation	1	25	26
**Reader 2**			
Positive/inconclusive radiograph interpretation	40	18	58
Negative radiograph interpretation	3	27	30
**Reader 3**			
Positive/inconclusive radiograph interpretation	41	12	53
Negative radiograph interpretation	2	33	35

When the radiographic interpretations were combined through majority vote method, 45/88 (51%) of lesions were assessed as aggressive/malignant, 31/88 (35%) as benign and 12/88 (14%) were assessed as inconclusive. This resulted in an overall sensitivity of 95% and specificity of 64%.

There was an overall moderate agreement between the readers as calculated by the kappa value. Using a weighted kappa value when combining the inconclusive final decisions and aggressive/malignant decisions as positive interpretations and benign interpretations as negative interpretations, there was a higher inter-observer agreement as there were only two variables with values bordering between moderate and substantial agreement (Table 4).

**TABLE 4:** Inter-observer reliability.

Rater 1	Rater 2	Kappa	LL 95%	UL 95%	Weighted kappa	LL 95%	UL 95%
Overall	-	0.43	0.33	0.53	0.57	0.36	0.74
Reader 1	Reader2	0.43	0.30	0.54	0.63	0.42	0.80
Reader 1	Reader 3	0.39	0.26	0.52	0.48	0.28	0.65
Reader 2	Reader 3	0.46	0.32	0.60	0.59	0.39	0.77

LL, lower limit; UL, upper limit.

Note: Variables included: Readers 1, 2 and 3.

**FIGURE 2:**
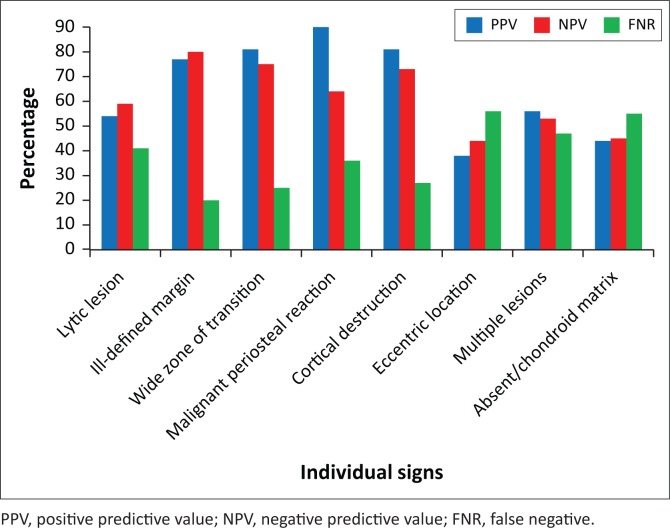
Positive and negative predictive values of individual signs used.

The PPV and NPV are presented in Figure 2. The four signs with the highest PPV and NPV were the same for all three readers and the majority vote. The order of decreasing PPV was malignant periosteal reaction (90%), cortical destruction (81%), wide zone of transition (81%) and ill-defined margin (77%). The signs with the highest NPV were ill-defined margin (80%), wide zone of transition (75%), cortical destruction (73%) and malignant periosteal reaction (64%). (These are referred to as ‘major signs’ from this point.)

Calculating pooled results did not lead to higher values because of inter-rater variability. The presence of all four major signs was associated with 100% PPV in predicting malignancy or infection.

## Discussion

In our study the percentages of aggressive lesions (including both malignant tumour and osteomyelitis) and benign bone tumours was similar ( 49% vs. 51%). Of the aggressive lesions, 16% were attributable to infection. Osteosarcoma was the most common malignant lesion (25.6%), and non-aggressive giant cell tumour (15%) and osteochondroma (13%) were the two most common benign bone tumours.

These findings are consistent with other studies performed on the African continent. In a large Nigerian study, Obalum et al. found that of the reviewed biopsies, 54% were benign and 46% malignant.^23^ The most common benign lesion in their series was osteochondroma (15%), with the most common malignant lesion being osteosarcoma (27%). The mean age of patients in their study was 25 years and the peak incidence of biopsied bone lesions occurred in the third decade. Another Nigerian study found 30% of biopsies to be benign, 49% histologically malignant (including 28% metastatic deposits and 17% osteosarcomas). Nine percent of biopsies in this series had an inconclusive histology.^22^ The higher incidence of infective lesions and relatively higher rate of osteogenic sarcoma compared with our study are explained by the fact that the mean age of their sample was 32 years and included a higher proportion of children. Our study did not include patients younger than 13 years as they are treated at an affiliated dedicated paediatric institution. The higher prevalence of metastases could be because of the inclusion of rib, vertebra and pelvic lesions.

In a recent series in India, Laishram et al. also found that the most common malignant tumour was osteosarcoma (11%), with osteochondroma (22%) being the commonest benign bone lesion. Their study revealed a surprisingly high prevalence of chronic osteomyelitis comprising 37% of their aggressive appearing lesions, resulting in a disproportionately high rate of malignant and infective lesions (58%).^21^ Our study had a significantly lower prevalence of osteomyelitis (16%), again probably because of the fact that osteomyelitis is more common in children.^29^ Another explanation could be that the diagnosis of chronic osteomyelitis at our institution is based largely on clinical, biochemical and microbiological evaluation rather than on imaging and biopsy.

Similar results have been reported in the developed world. In a study of more than 100 biopsy specimens, Jelinek et al. had a significant higher percentage yield for malignant histology (70% vs. 49%). The proportion of the most common malignant and benign tumours was similar to our study, with osteosarcoma being 18% and non-aggressive giant cell tumour 15%. The mean age in their study population was 38 years.^17^ The differences could be explained by the fact that their study exclusively analysed the histology of primary bone tumours and excluded metastases, infections, and inflammatory and metabolic diseases.

In our series, we described 5/88 (6%) cases with pathologically confirmed osseous tuberculosis (TB). Extra-pulmonary TB is seen in 1% – 3% of patients with TB in the developed world and approximately 10% in endemic countries such as South Africa. Moreover, the risk of TB is 20% – 37% higher in patients with human immunodeficiency virus (HIV). The relatively low rate of skeletal TB in our series could be because skeletal TB is rare when compared with pulmonary TB and TB lymphadenitis. Most osseous TB (50%) affects the spine whilst 15% of cases present as septic arthritis of the hip. Spinal biopsies and joint aspirates were not included in our series. Finally, TB of the spine and large joints is more common in children and young adults, and patients aged less than 13 years were excluded from our series.^29,30,31^

The kappa value of 0.61 for radiographic interpretation and final histology indicates substantial agreement, with an overall sensitivity of 80%. This finding is low when compared with similar studies. Vijayaraghavan et al. also correlated histological diagnosis with radiological interpretation based on Lodwick et al.’s method of classification and calculated an 80% case correlation.^13,25^ The high correlation between imaging and final diagnosis in their study could be attributed to a larger sample size and the fact that they reviewed clinical data, radiographs, CT and MRI and had discussions on the cases. Negash et al. reported a case correlation of 84% with a kappa agreement of 0.82 which could be because of the inclusion of MRI findings and consensus decision-making at combined clinical and radiological incorporation meetings.^26^

There was a high rate of ‘inconclusive’ interpretations by the readers, where radiographs were not convincing for aggressive or non-aggressive findings. Possible reasons for this include unwillingness to commit without cross-sectional imaging, absence of clinical history for patients aged more than 40 years and lack of awareness that the lesions were biopsied. This could be explained by the moderate inter-reader agreement. When the malignant/aggressive interpretations and inconclusive interpretations were combined as *positive* findings versus the benign radiographic findings as *negative* findings, the inter-observer agreement improved to 0.48–0.63, indicating moderate to substantial agreement between the readers.

Using set criteria, sensitivity was high (ranging between 93% and 98%) in correctly diagnosed malignant/infective lesions on radiographs. When using the ‘majority vote’, the sensitivity was 95%, with only two infective lesions that would have been missed/misinterpreted. There was no statistically significant difference between the sensitivity of individual readers and the pooled sensitivity using the majority vote method. This suggests an understandable reluctance to categorically call a lesion benign, given the serious implications of missing a malignancy or infection.

Reader specificity in the current study ranged from 53% to 73%, averaging to 64% with the ‘majority vote’. The true specificity could not be calculated because the majority of bone lesions with benign features are not biopsied. Another explanation for low specificity was the frequent (up to one-third of cases) selection of ‘inconclusive’ option.

Assessing a single radiological sign has some value in calculating PPV and NPV, with high PPV for malignant tumours and infection in the presence of an ill-defined margin, a wide zone of transition, malignant periosteal reaction and cortical destruction. However, 20% of these lesions yielded benign histology. The overall low PPV and NPV in the other four radiological signs (‘lytic lesion’, ‘eccentric location’, ‘multiple lesions’ and ‘absent or chondroid matrix’) could be explained by the fact that not all of these eight radiographic signs are necessarily present in an aggressive bone lesion, benign lesions could mimic aggressive lesions, malignant lesions could have varying appearances, and lesion matrix is more useful for guiding differential diagnosis than lesion aggressiveness.^7,12^

More importantly, the PPV for malignancy or infection increased proportionally with an increase in the number of positive radiological signs. If three signs were present, then PPV was 97%. Where there were four major signs, the PPV was 100%. Unfortunately, the absence of any of the major signs did not exclude malignancy or infection but had a combined yield of 10% for malignant bone tumour and infection.

## Implications, limitations and future applications

In this study where data were obtained from a tertiary hospital having a specialised oncology clinic, with experienced orthopaedic surgeons and musculoskeletal radiologists, half of the biopsied lesions were benign. Delay in diagnosis and treatment must be balanced against the financial implications of time-off work, loss of income and surgically related morbidity and mortality that may result from unnecessary surgical treatment.

The study is limited by its small sample number. Although the National Health Laboratory stores the histological results of previous 5 years, the hospital’s picture archiving and communication system (PACS) was introduced only in 2012, allowing review of digital radiographs of only 3 years. Printed radiographs were not accessible and were excluded from this study. Additionally, malignant bone lesions are relatively uncommon and copies of radiographs of patients referred for biopsy from outside institutions were not universally available for review. The study group is heavily skewed to include only those patients whose radiographs demonstrated lesions with aggressive imaging features, and also excluded children aged less than 13 years, thus underestimating true incidences in the referral region.

The current study only evaluated radiographic interpretation and further research could have included the contribution of MRI in improving the sensitivity and specificity of identifying malignant bone lesions. Additionally, a combined weekly meeting at our institution would have helped aiding in bone tumour characterisation, improving registrar training and ultimately leading to better management of patients with bone tumours. Similar studies may also include the classic benign ‘leave-alone’ or ‘do not touch’ bone lesions for assessment of readers’ knowledge in identifying benign bone lesions.

A larger review study is recommended to assess radiographic features and incorporate a scoring system. This could lead to further categorisation of bone tumours and ultimately to improved confidence of clinicians and radiologists in their assessment and recommendations on follow-up and need for further imaging investigations or biopsy.

## Conclusion

The study findings concur with other reported studies from developed and developing countries. The demonstration of high sensitivity in diagnosing primary malignant bone tumours using an established systematic review schema when interpretating radiographs confirms their usefulness as a screening tool. Contrasting this, the low specificity may be attributed to the fact that benign bone lesions often have imaging findings that mimic aggressive lesions. We found that the absence of any of the four ‘major radiological signs’ had a low yield (10%) for malignancy and the presence of all 4 major signs had a 100% yield for malignancy or osteomyelitis.

Even with experienced readers, the diagnosis of primary malignant tumours could be difficult and the fear of missing a malignancy often results in a large number of unnecessary biopsies. A combined clinico-radiological and histopathological approach with regular follow-up and MRI in selected cases could allow more accurate diagnosis and improved patient management.
